# Cryo‐electron microscopy visualization of a large insertion in the 5S ribosomal RNA of the extremely halophilic archaeon *Halococcus morrhuae*

**DOI:** 10.1002/2211-5463.12962

**Published:** 2020-09-17

**Authors:** Madhan R. Tirumalai, Jason T. Kaelber, Donghyun R. Park, Quyen Tran, George E. Fox

**Affiliations:** ^1^ Department of Biology and Biochemistry University of Houston TX USA; ^2^ National Center for Macromolecular Imaging Baylor College of Medicine Houston TX USA; ^3^ Present address: Rutgers New Jersey Cryo‐electron Microscopy & Tomography Core Facility Institute for Quantitative Biomedicine, Rutgers The State University of New Jersey Piscataway NJ USA; ^4^ Present address: Department of Microbial Pathogenesis Yale University New Haven CT USA

**Keywords:** accretion model, archaea, expansion sequences, insertion sequences, ribosomal RNA

## Abstract

The extreme halophile *Halococcus morrhuae* (ATCC^®^ 17082) contains a 108‐nucleotide insertion in its 5S rRNA. Large rRNA expansions in Archaea are rare. This one almost doubles the length of the 5S rRNA. In order to understand how such an insertion is accommodated in the ribosome, we obtained a cryo‐electron microscopy reconstruction of the native large subunit at subnanometer resolution. The insertion site forms a four‐way junction that fully preserves the canonical 5S rRNA structure. Moving away from the junction site, the inserted region is conformationally flexible and does not pack tightly against the large subunit. The high‐salt requirement of the *H. morrhuae* ribosomes for their stability conflicted with the low‐salt threshold for cryo‐electron microscopy procedures. Despite this obstacle, this is the first cryo‐electron microscopy map of *Halococcus* ribosomes.

## Graphical abstract

The ribosome is an ancient and universal ribonucleoprotein molecular machine, with fingerprints of ancient and recent insertions seen in its structure. The extremely halophilic archaeon *Halococcus morrhuae* (ATCC^®^ 17082) possesses an unusual insertion in the 5S rRNA, almost doubling its length. Cryo‐EM reveals a four‐way junction at the insertion site, accommodating the insert without disrupting the canonical 5S structure.

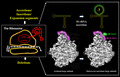

**Abbreviations**
cryo‐EM: cryo‐electron microscopy
LSU: large subunit
rRNA: ribosomal RNA

Single‐molecule fluorescence resonance energy transfer (smFRET), and crystallographic and cryo‐electron microscopy (cryo‐EM) techniques have been used to obtain high‐resolution structures of ribosomes and their components at various stages of translation [1, 2, 3, 4, 5, 6]. These studies have been instrumental in our understanding of ribosome evolution.

Ribosomal diversity has emerged in part by stepwise addition of insertion sequences that ‘grow’ the ribosome outward, from the common core to the large ribosomes of extant complex metazoans [7, 8]. In some instances, it has been possible to recognize that what is now a common part of the rRNA likely began as an insertion [7, 9, 10, 11]. Such insertions have been used to deduce the relative age of various regions in the rRNAs [7]. Insertions typically are accommodated in the ribosome by forming a three‐ or four‐way junction with negligible perturbation of the parental helix [12].

5S rRNA as an integral part of the ribosomal large subunit LSU [13] may function as a link between the peptidyl transferase center and GTPase center of the ribosome via loop D (Fig. 1B) [14, 15, 16, 17, 18, 19], as well as through its interacting proteins uL5, uL18, and L25 [20, 21, 22, 23]. Proper maturation of the 5S rRNA precursor is essential for assembly of the central protuberance [13, 19, 24, 25]. Of special interest here is the 5S rRNA isolated from *Halococcus morrhuae* (ATCC^®^ 17082), which contains a large 108 nucleotide insertion [26] that almost doubles the size of the RNA (Fig. 1). The insertion is found between *H. morrhuae* residues 104 and 105 [26], which corresponds to residues 108 and 109 in the universal bacterial 5S rRNA numbering system [27, 28]. All members of the genus *Halococcus* that have been examined to date have this insertion [29, 30]. Although insertion/expansion sequences of varying size are frequently seen in the large rRNAs of eukaryotes [31, 32], similar large insertions have typically not been described in archaea [12]. Exceptions occur in the Asgard archaea [33]. In bacteria, there has been at least one notable instance, wherein an insertion known as the ‘steeple’ (causing a small‐subunit‐dependent conformational change) has been described in *Mycobacterium smegmatis* [34, 35]. The fact that the *H. morrhuae* insert is almost as large as its parent rRNA raises the question of whether the presence of the insert may impact the canonical 5S structure. In order to address this issue, cryo‐EM was used to characterize the 3D structure of the native large subunit of *H. morrhuae* (ATCC^®^ 10782).

**Fig. 1:**
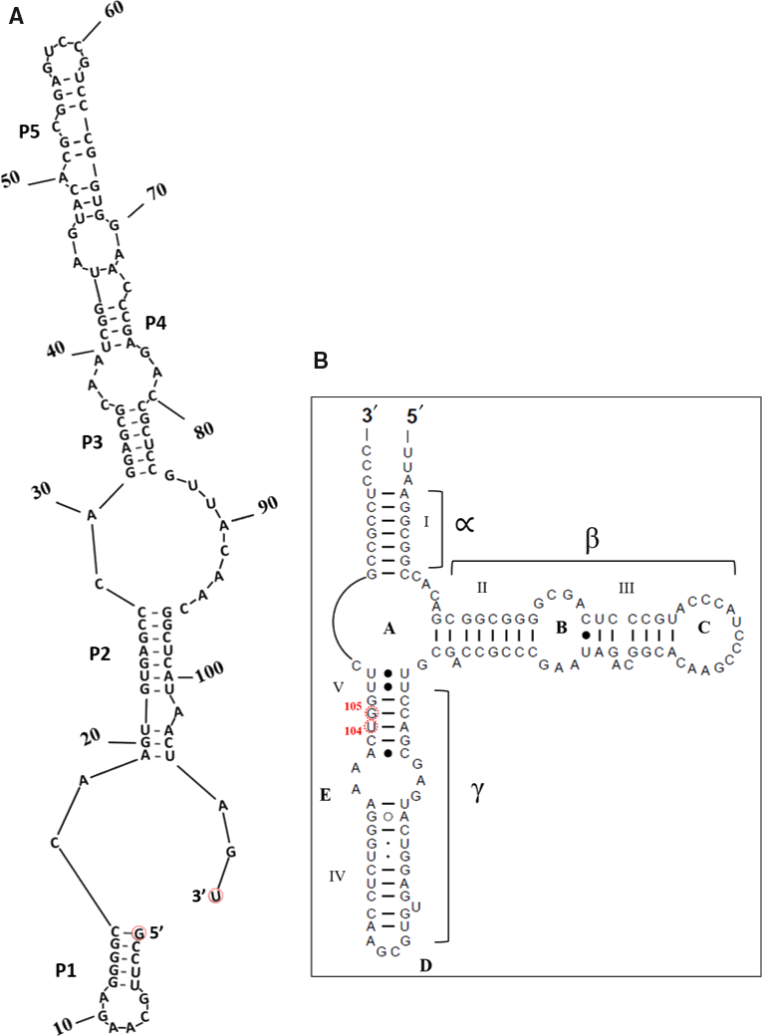
An unusual 5S rRNA insertion. A schematic diagram showing (A) a possible secondary structure of the insert as predicted by mfold [50] and RNAstructure [51] and (B) the usual 5S rRNA secondary structure model (available at RNAcentral (https://rnacentral.org/) and the Comparative RNA Web (CRW) Site( http://www.rna.ccbb.utexas.edu)) [52, 53] and modified; the insert is between positions U104 and G105 as per *Halococcus morrhuae* 5S rRNA numbering. The equivalent positions in *Haloarcula marismortui* are C108 and G109. What would be the 5′ and 3′ ends of the insert if it were an independent RNA are indicated in (A).

## Results and Discussion

Secondary structure prediction suggests several alternative structures for the insert. The insert structure with the most negative delta G (−44.8 kcal·mol^−1^) is shown in Fig. 1A. In this predicted structure, the insert alone would form an elongated region with five helical regions, each consisting of four or more standard base pairs. From a transcriptional perspective, synthesis would progress from the 5′ end of helix I, which would then be completed before the other helices are even started. The 5S rRNA beta and most of the gamma stems are also complete (Fig. 1B), before the insert is made, which likely would help minimize misfolding. This would result in a small structural domain consisting of helices I, II, III, and IV and a large domain encompassing the rest of the insert. The predicted helices in the insert are consistently separated by bulge loops that likely allow changes in orientation such that the unpaired segments of the RNA likely act as hinge regions to facilitate dynamics.

Large ribosomal subunits from *H. morrhuae* ATCC^®^ 17082 were purified and imaged by cryo‐EM. This allowed us to obtain a 3D model of the 5S rRNA in the context of the intact large subunit. In pilot cryo‐EM experiments on *H. morrhuae* ribosomes, the quality was poor, likely due to their lack of stability at low‐salt concentrations [36]. Therefore, ribosomes were maintained in the high‐salt buffer A until vitrification. The salt concentration was then lowered with an on‐grid washing procedure. Reconstructions to 12 Å were obtained from an initial low‐magnification dataset of ~ 15 000 particles. This revealed density protruding from the expected site of the 5S rRNA. This protrusion is not present in other known LSU structures such as that of *Methanothermobacter thermautotrophicum* (Fig. 3C) (PDB ID 4ADX) and *Haloarcula marismortui* [37, 38]. Docking known 5S rRNA structures into the map showed that the site of the protruding density matches, to the nucleotide, the expected site of the insertion assuming no insertion‐induced changes in the 5S rRNA (Figs 1, 3). The protruding density fades and broadens as the distance from the core increases, which could indicate flexibility of the looped‐out region.

**Fig. 2:**
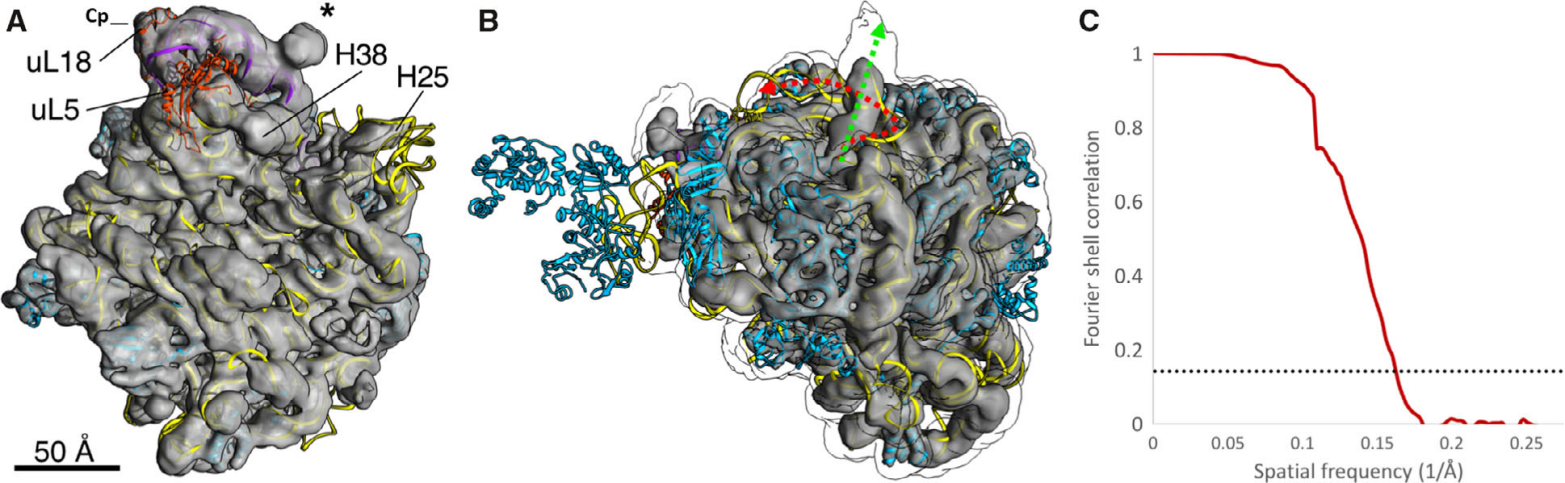
Cryo‐EM reconstruction of the large subunit of *Halococcus morrhuae*. (A) The large subunit of *Haloarcula marismotui* (PDB: 1NJI) [49] is rigidly docked in the density obtained from 3D cryo‐EM reconstruction; 5S rRNA (purple), 23S rRNA (yellow), and uL5 and uL18 (orange‐red), and selected ribosomal other proteins (cyan) are shown; the insert is marked by *; Cp—central protuberance. The scale bar indicates 50 Å. (B) A view of the large subunit with the best‐available crystal structure docked in (PDB: 4V9F) [48] rotated to focus on the discordance in the helix 25 path (red arrow summarizing the crystal structure and green arrow summarizing the cryo‐EM structure). The cryo‐EM density at lower isosurface threshold is shown as an outline around the map, illustrating the continued projection of the helix. The P stalk can be seen on the left edge; though the P stalk proteins L11, L12, and L10e are visualized in the crystal structure of *Haloarcula marismotui*, they are not seen in this cryo‐EM map. Scale is identical to panel A. (C) Plot of the consistency of the two half‐maps after tight masking and mask correction. The 0.143 ‘gold‐standard’ cutoff is indicated as a dashed line.

**Fig. 3:**
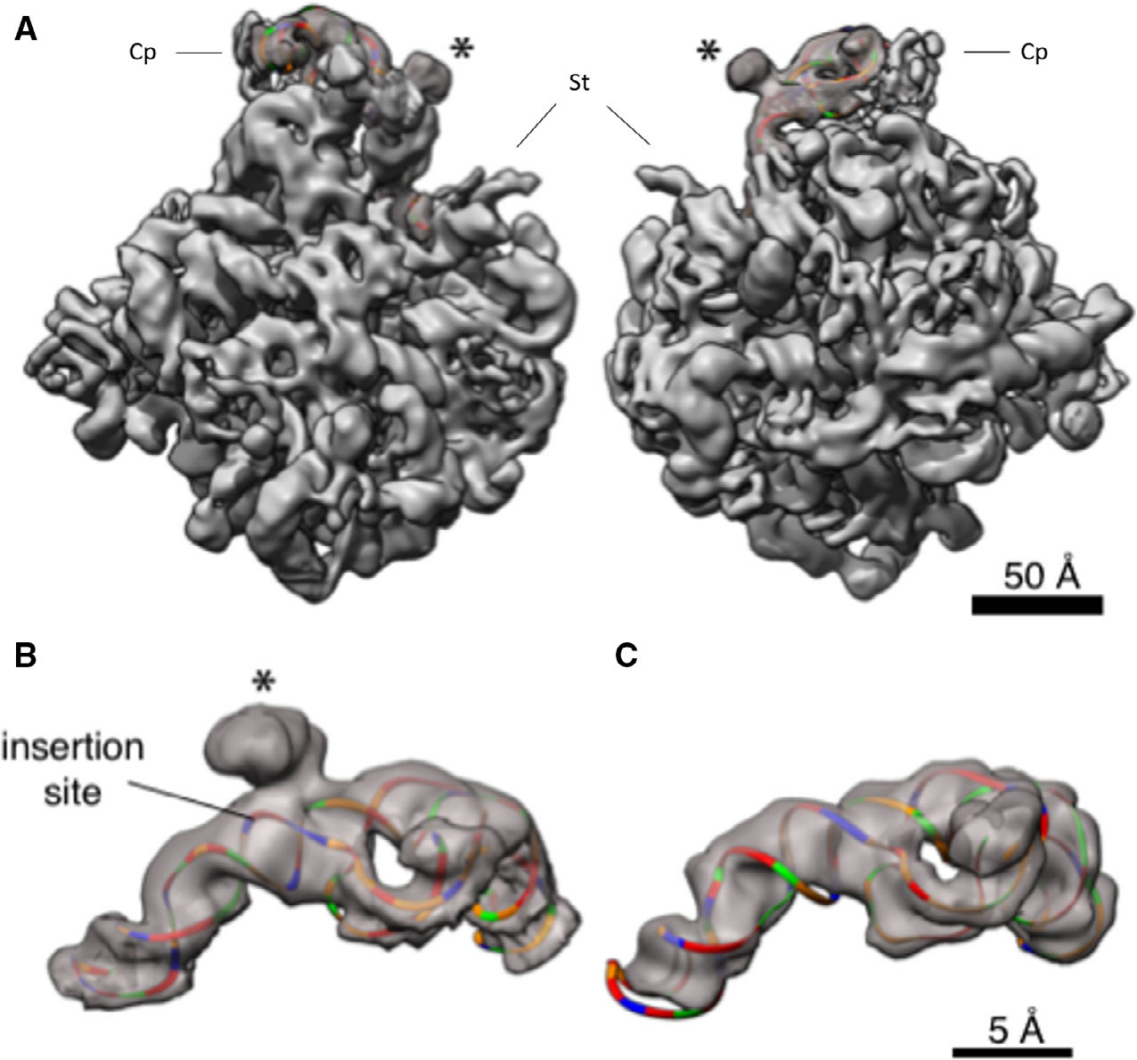
3D structure of the 5S rRNA. (A) The large subunit of *Haloarcula marismotui* (PDB: 1NJI) [49] is docked in the density (left, view from the 30S side; right, view from the solvent side of the 50S subunit); only the 5S rRNA is shown colored, and the insert is marked by *. Coloring is by nucleotide: adenines, green; cytosines, orange; guanines, red; and uracils, blue. Cp—central protuberance; St—stalk. The scale bar indicates 50 Å. (B) The section of the map corresponding to the 5S rRNA is cropped out for visualization. The *Haloarcula marismortui* 5S rRNA was mutated to match the sequence of the *Halococcus morrhuae* 5S, except that the insert (whose secondary structure is not known) was omitted, and rigidly docked inside the density. The insertion site is marked by *. The scale bar indicates 5 Å. (C) The section of the map corresponding to the 5S rRNA is cropped out of the published, 6.6 Å *Methanothermobacter thermautotrophicus* cryo‐EM map and associated atomic model (EMD‐2012, PDB 4ADX) [37] and colored as in (B). This and all other homolog maps lack the protruding lump of density seen in (B). Scale is identical to panel B.

Using freshly prepared LSUs, the imaging was repeated at higher magnification and with a larger dataset of over 100 000 particles. These data were reconstructed to a nominal resolution of 6.4 Å (EMD‐21670) (Fig. 2, Movie S1). Real‐space local resolution quantification with resmap [39] also estimates a median resolution of approximately 7 Å and modal resolution around 6 Å (Movie S2). RNA and protein secondary structural elements are clearly seen in the interior of the particle (Fig. 2A). The density seen for the insertion still projects off of base 108 of the 5S rRNA (Fig. 3, Fig. S1), but it is poorly resolved and only large enough to accommodate around 40 of the 108 inserted nucleotides as a continuous double helix. Such density is not observed in cryo‐EM maps of any published ribosome, such as *Methanothermobacter thermautotrophicus* [33] (Fig. 3C). The density suggests helices extending at 120° and 210° relative to the parent helix in a four‐way junction (Fig. 3B). The junction resembles the specificity domain four‐way junction of RNase P [40], which is the type specimen of four‐way junction family ‘H’ [41]. The fact that the bulge does not accommodate all of the insert and that an abrupt cutoff is not seen but rather a gradual broadening and fading of the density suggests that the insert may ‘wave’ like a flag in the wind.

At low isosurface thresholds, some density can be seen stretching from the insertion site to helix 38 near *Escherichia coli* positions 970–990 at the subunit interface. Helix 38 makes a lateral contact with helix 84 in the 23S rRNA (Fig. S2) and houses the A‐site finger that aids in positioning the tRNA in the A‐site [42, 43]. The faint density does not conflict with the locations of the nearby ribosomal proteins uL18 and uL5 (Fig. 2A). uL5 density is fainter than uL18 density, potentially indicating partial occupancy in this dataset. However, from these maps we are not able to determine whether the faint density stretching from the insertion site to helix 38 represents genuine, flexible 5S rRNA density or noise.

No other post‐LUCA ribosomal insertion has almost doubled the size of a rRNA. Yet, even this large insertion obeys the general rules that have been proposed for ribosomal accretion [12] in that it leaves a typical insertion fingerprint and causes negligible rearrangement of nucleotides in the parent helix. The apparent flexibility of the inserted nucleotides is atypical, although there are precedents, such as the flexibility of ribosomal RNA elements in *Staphylococcus aureus* [44], *Trypanosoma brucei* [45], and plant mitoribosomes [46]. The observed flexibility could be an intrinsic feature of this rRNA insertion reflecting its age or function, or the insertion might assume a homogeneous conformation in the presence of a binding partner [34, 35, 47]. The conformational landscape and the evolutionary history of the inserted region within the *Halococcus* 5S rRNA thus warrant future study.

Aside from the 5S rRNA, comparisons of the *H. morrhuae* large subunit map obtained herein to the crystal structures of *H. marismortui* [48, 49] reveal minor differences at the subunit outer surface. For example, in this map, but not in the crystals, the end of helix 38 is seen (Fig. 2A). On the opposite side of the 5S rRNA insertion site is helix 25. Whereas in the crystal structure helix 25 wraps against the ribosome, in solution it extends outward. Visualized at a stringent isosurface threshold, the helical axis is directly observed and points outward, while at a liberal threshold, the outline of more distal density continues along the same vector (Fig. 2B). Therefore, the flipped bases such as 578–580 in crystal structures from *H. marismortui* are likely crystal packing artifacts. In cells, these bases would be solvent‐exposed rather than packed against the ribosomal surface.

Ribosome binding in haloarchaea has been reported to require molar concentrations of salt [36]. This implies that the *H. morrhuae* ribosomes require a high‐salt buffer to maintain ribosome stability, which represents a biochemistry bottleneck for cryo‐EM procedures. Furthermore, analogous to the flexible parts of ribosomes in both bacteria and eukarya [44, 45, 46], the inability to achieve higher resolution may be partly attributable to the flexibility of the 5S rRNA insert.

## Materials and methods

### Computational analysis

A prediction of the insert's possible secondary structure was made using mfold [50] and RNAstructure [51]. Several possibilities were found. The structure with the lowest free energy is shown in Fig. 1A. The 5S rRNA secondary structure model without the insert available at RNAcentral (https://rnacentral.org/) and the Comparative RNA Web (CRW) Site (http://www.rna.ccbb.utexas.edu) [52, 53], was modified and is shown in Fig. 1B.

### Medium, culture/strain, and maintenance

*Halococcus morrhuae* (Farlow) Kocur and Hodgkiss (ATCC^®^ 17082^™^) was obtained from the ATCC and maintained on 25% NaCl media containing casamino acids, and other salts [29].

### Cell growth and lysis for extraction of the ribosomes

*Halococcus morrhuae* cells were grown on a large scale using a minimum of 1 L of culture broth, at 37 °C, 200 r.p.m. The cells were harvested during mid‐log phase and suspended in buffer A (3.4 m KCl, 60 mm Mg (OAc), 30 mm Tris/HC1, 7 mm 2‐mercaptoethanol, pH 7.6) [54]. Suspended cells were passed through a French press multiple times. The cell lysates were treated with RNase‐free DNase (Promega Corp, Madison, WI) for 30 min, following which the lysate was processed through ultracentrifugation as described [54, 55]. As a first step, the cell lysates were centrifuged at 30 000 ***g*** for 30 min to remove the debris in a SW28 rotor using a Beckman Coulter ultracentrifuge (Beckman Coulter, Brea, CA). The upper two‐thirds of the supernatant was collected and centrifuged in a SW28 rotor, at 20 000 r.p.m (55,000 g). for 17 h. In one previous study involving ribosome isolation from *H. marismortui*, the red gelatinous material, presumably containing the pigments, that was found atop the ribosome pellet, was removed physically [56]. However, in the case of *H. morrhuae*, the ribosome pellet was found to be mixed with pigments, which could be an impediment to cryo‐EM reconstruction. Ribosomes have been shown to be precipitated by the addition of acetone or ethanol [57]. The ribosomes were separated from the pigments, by treating the ribosome pellet with acetone (100%) in a 50‐mL tube. The tube was intermittently inverted for 15–20 min to allow the separation of the pigments into acetone. The acetone phase was then carefully removed using a sterile pipette. The ribosomal pellet was air‐dried and suspended in buffer A. To obtain the ribosomal subunits, ribosomes in buffer A were diluted approximately 10‐fold with dissociation buffer (2.7 m KCl). Next, they were separated for 15 h by zonal centrifugation using a linear, 6–36% (w/v), sucrose gradient in a SWTi 55 rotor, at 22 500 r.p.m. (100 000 ***g***) [54]. The subunits were then pooled separately from the fractions, washed 2–3 times with buffer A to remove the sucrose, and finally stored in buffer A at −80 °C.

### Cryo‐electron microscopy

Ribosomes were vitrified and imaged by cryo‐EM. Preliminary images in buffer A presented low contrast, presumably attributable to the high‐salt concentration. We therefore applied an on‐grid washing procedure. Quantifoil R2/1 grids were plasma‐cleaned for 5 s using a Solarus plasma cleaner (Gatan, Pleasanton, CA). In the humidity‐controlled chamber of the Vitrobot Mark IV, 2 μL sample was applied to the grid, and then 6 μL of distilled water was applied to the reverse face of the grid and immediately blotted. The droplet of water on the reverse face was observed to travel through the grid and mainly pool on the plasma‐cleaned face. A typical blot time for grids used in this study was 3 s.

Micrographs were collected using a JEM‐3200FSC electron microscope (JEOL USA, Peabody, MA) with K2 Summit direct electron detector (Gatan). The final dataset was imaged at 1.97 Å·pixel^−1^, nominally 20 000× magnification, with a total exposure of 26e^−^·Å^−2^ over 8 s. 1000 fields of view were collected over the course of two imaging sessions, of which 795 were accepted for processing. Subnanometer reconstructions were obtained using both Relion‐2 and cryoSPARC v1 (Structura Biotechnology Inc., Toronto, Canada). Data were traceably archived using the EMEN2 object‐oriented database [58] until processing methods improved. After further developments in cryo‐EM software, the dataset was reprocessed. Relion‐3.1 was used for motion correction [59], CTF fitting, and particle extraction. cryolo [60] was used for neural‐net‐based particle picking. 140 444 particles were picked. The subset of particles exhibiting neither aggregation nor self‐self‐interaction was manually extracted for reconstruction. 2D classification of a subset of 99 337 particles, *ab initio* reconstruction, and nonuniform refinement were performed in cryosparc v2 (Structura Biotechnology Inc.) [61]. More stringent classification to a 69 042‐particle subset did not improve the structure, nor did particle‐subtracted refinement of the 5S rRNA alone. Fourier shell correlations were measured between half‐maps at the 0.143 resolution (Fig. 2C). Half‐maps were independent beyond 20 Å.

For structure interpretation, known structures from the related halophilic archaeon *H. marismortui* [48, 62, 63] and the relatively distant methanogenic archaeon *M. thermautotrophicus* [37] were docked onto the electron density of the *H. morrhuae* ribosome particles. Maps were rendered using ucsf chimera or uscf chimera x [64]. Segmentation of the map to isolate the 5S region (Fig. 3B,C) was performed in Chimera by using the atomic models of archaeal ribosomes and applying the ‘Split Map’ command.

## Conflict of interest

The authors declare no conflict of interest.

## Author contributions

GEF conceived the work; GEF, MRT, and JTK planned the work; MRT maintained the culture, grew the cells, extracted, and purified the ribosomes; JTK and DRP performed cryo‐EM experiments; MRT and JTK constructed figures; MRT, JTK, QT, and GEF analyzed the structure; and all authors co‐wrote the paper.

## Supporting information


**Fig. S1.** Closeup view of the insert location with the large subunit of *Haloarcula marismotui* (PDB: 1JJ2) [38] docked in the cryoEM density; the bases (C108 and G109) between which the insertion occurs are marked; the insert (blob) is colored in green outline; the 23S rRNA is in yellow, 5S rRNA is in blue, uL18 is in magenta, uL5 in red.
**Fig. S2.** Zoomed‐in top view of the 50S subunit. Features surrounding the 5S rRNA extension (green) are labeled and the canonical 5S rRNA is outlined in red.

**Movie S1.** Overall cryo‐EM map of the large subunit of the *Halococcus morrhuae* ribosomes, at different thresholds, showing the insert, with the large subunit of *Haloarcula marismotui* (PDB: 1NJI) [49] docked in the cryo‐EM density.

**Movie S2.** ResMap local resolution estimation, measured by the method of two half‐maps, is displayed as colored regions overlaid onto the final map.

## Data Availability

The cryo‐EM reconstruction of the *H. morrhuae* (ATCC^®^ 17082) large subunit is deposited as EMD‐21670. Data will be available from the corresponding author upon reasonable request.
