# Supplementary material for: Cryo‐electron microscopy visualization of a large insertion in the 5S ribosomal RNA of the extremely halophilic archaeon Halococcus morrhuae
Source: FEBS Open Bio. 2020 Sep 17;10(10):1938–46. doi: 10.1002/2211-5463.12962 (PMC7530397; doi:10.1002/2211-5463.12962)
Supplement: Supplementary file 1 — Fig. S1. Closeup view of the insert location with the large subunit of Haloarcula marismotui (PDB: 1JJ2) [38] docked in the cryoEM density; the bases (C108 and G109) between which the insertion occurs are marked; the insert (blob) is colored in green outline; the 23S rRNA is in yellow, 5S rRNA is in blue, uL18 is in magenta, uL5 in red. Fig. S2. Zoomed‐in top view of the 50S subunit. Features surrounding the 5S rRNA extension (green) are labeled and the canonical 5S rRNA is outlined in red. [file FEB4-10-1938-s001.docx]

**Supplementary File**

**Supplementary Figure 1.**

**
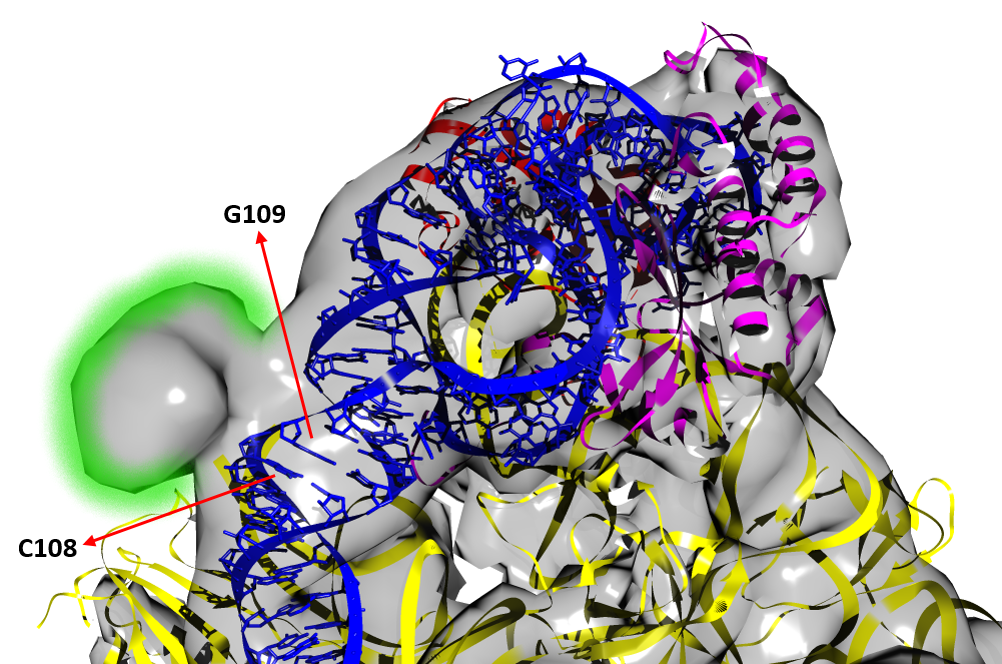
**

Closeup view of the insert location with the large subunit of *Haloarcula marismotui* (PDB: 1JJ2) [38] docked in the cryoEM density; the bases (C108 and G109) between which the insertion occurs are marked; the insert (blob) is colored in green outline; the 23S rRNA is in yellow, 5S rRNA is in blue, uL18 is in magenta, uL5 in red.

**Supplementary Figure 2.**

**
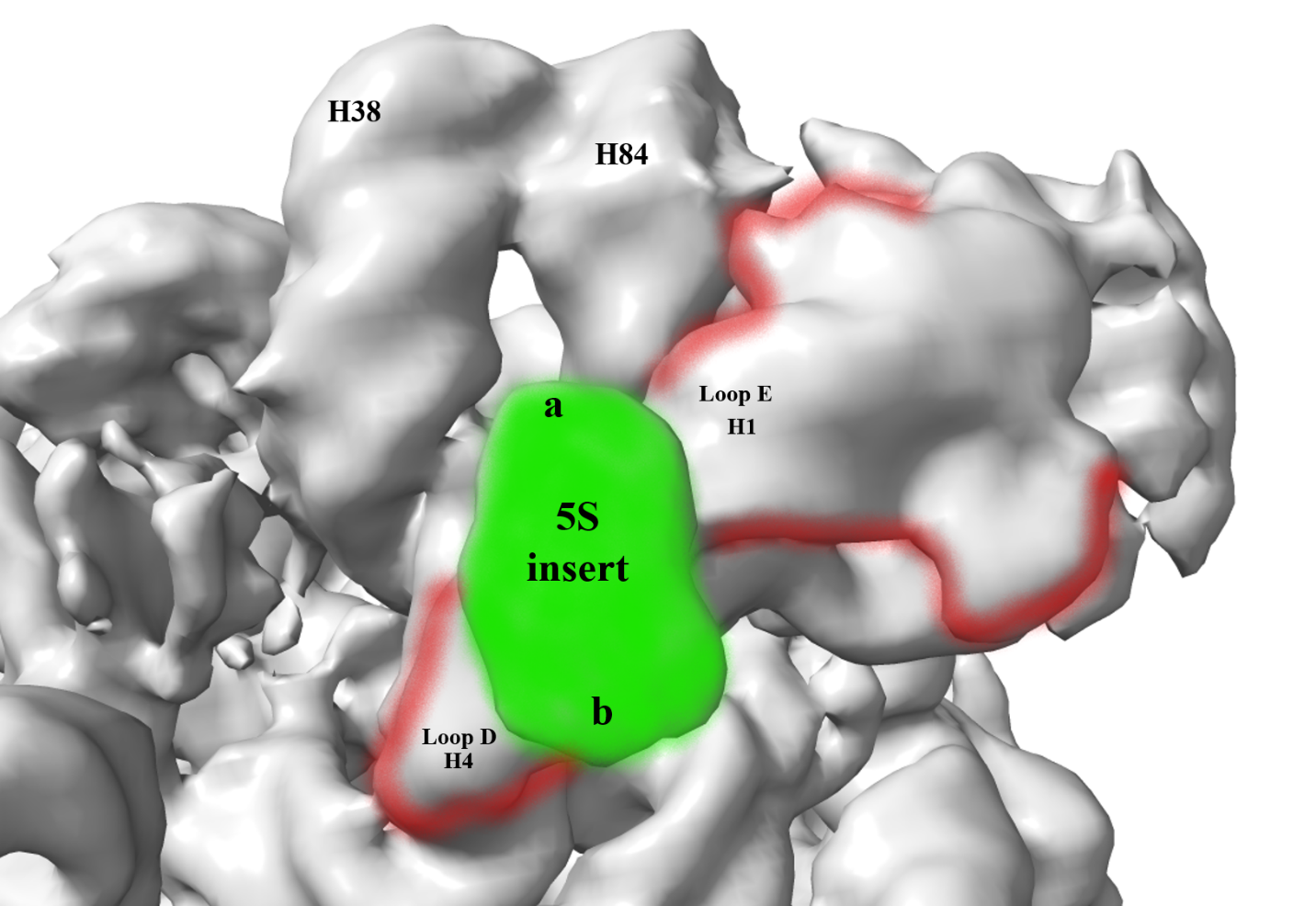
**

Zoomed-in top view of the 50S subunit. Features surrounding the 5S rRNA extension (green) are labeled and the canonical 5S rRNA is outlined in red.
